# Poor Sleep Is a Risk Factor for Low-Back Pain among Healthcare Workers: Prospective Cohort Study

**DOI:** 10.3390/ijerph17030996

**Published:** 2020-02-05

**Authors:** Jonas Vinstrup, Markus D. Jakobsen, Lars L. Andersen

**Affiliations:** National Research Centre for the Working Environment, DK-2100 Copenhagen, Denmark; mdj@nfa.dk (M.D.J.); lla@nfa.dk (L.L.A.)

**Keywords:** healthcare, nurses, occupational injuries, low back pain, workplace

## Abstract

This study aimed to investigate the association between poor sleep and risk of low-back pain (LBP) in healthcare workers. Using a prospective cohort design with 1-year follow-up, a total of 1955 healthcare workers (60% nurses) from 389 departments at 19 hospitals responded to questionnaires containing items related to lifestyle, health, and working environment. Associations between sleep scores (0–100) at baseline and LBP intensity (0–10) at follow-up were modelled using cumulative logistic regression accounting for clustering at the department level and adjusted for lifestyle and psychosocial confounders. In the full population of healthcare workers, 43.9% and 24.4% experienced moderate and poor sleep, respectively. In the fully adjusted model with good sleep as reference, moderate, and poor sleep increased the risk of LBP at follow-up, with odds ratios (OR’s) of 1.66 (95% confidence interval (CI) 1.35–2.04) and 2.05 (95% CI 1.57–2.69), respectively. Three sensitivity analyses including healthcare workers free from LBP, nurses, and nurses free from LBP at baseline, respectively, yielded similar results. In conclusion, poor sleep constitutes a potent risk factor for LBP among healthcare workers. The presented results provide strong incentives to evaluate and weigh current prevention policies against an updated biopsychosocial framework.

## 1. Introduction

Sleep constitutes an equally complex and vital biological function, with the behaviorally-driven goal to maintain homeostasis across multiple physiological systems: It is essential for recuperation of physical and psychological stressors, learning, physical performance, cognition, emotional modulation, brain plasticity memory encoding, and consolidation, as well as overall health of the mammalian organism [1,2,3,4]. Furthermore, poor sleep poses a well-known and potent risk factor for a multitude of physical and physiological ailments, including obesity, dementia, diabetes, widespread and chronic pain, as well as all-cause mortality [5,6,7,8,9,10].

In mechanical, interventional and epidemiological studies alike, considerable effort has been devoted to progressively shed light on the relationship between sleep and pain [8,11,12,13,14,15,16,17]. For example, a recent meta-analysis with pooled estimates from 37 studies using polysomonography in populations with persistent pain, reported a 72% prevalence of insomnia [18]. This interrelation between sleep and pain is echoed in recent reviews [8,13,19], which, in contrast to previous studies investigating (bi)directionality, highlights a more consistent unidirectional effect of poor sleep on pain exacerbation and vulnerability.

Despite uncertainty regarding underlying mechanisms, the link between sleep and pain has, therefore, been diligently established. Following this, populations exposed to work-related and environmental stressors (e.g., odd working hours, job dissatisfaction, psychosocial stress etc.) are, therefore, likely to be more vulnerable to pain due to not only the stressors themselves [20], but, perhaps more importantly, due to how they negatively affect sleep. Healthcare workers arguably constitute a subgroup of the working population especially prone to the detrimental effects of a notorious working environment; known to foster high levels of fatigue, psychosocial stress, perceived exertion and musculoskeletal pain [21,22,23]. Consequently, diminishing the tally of potent risk factors for developing/exacerbating pain in healthcare workers holds the potential to substantially enhance the quality of said working environment.

Previous research from our group reports cross-sectional associations between stress, pain and the outcome of poor sleep [24], but the prospective relationship between sleep and low-back pain (LBP) among healthcare workers is lacking in detail. Therefore, the primary aim of this study was to investigate associations between baseline sleep scores and risk of LBP at 1-year follow-up in a population of Danish healthcare workers. Additionally, sensitivity analyses were performed and included (1) female nurses, (2) female nurses pain-free at baseline as well as (3) healthcare workers pain-free at baseline.

## 2. Materials and Methods

In line with the proposed aim of this study, the analyses presented in this study make it possible to (1) investigate associations between sleep and LBP and (2) to identify potential differences between sub-groups of the working population (nurses) as well as between those with LBP and without LBP at baseline. Using a prospective cohort design with baseline- and 1-year follow-up questionnaires, this study therefore reports associations between sleep and LBP intensity. Specifically, we investigated the potential dose-response relationships between subjective ratings of sleep quality (poor, moderate or good) at baseline and risk of a 1-point increase in LBP intensity on the visual analog scale (VAS) at follow-up. We have previously reported prospective associations between patient transfers and risk of back injury in this population [25].

### 2.1. Study Design and Participants

The baseline questionnaire was sent to 7025 hospital workers from 389 departments at 19 hospitals in Denmark, of which 4151 (59%) responded to the full questionnaire. In this analysis, we include nurses, healthcare assistants, nurses aids, physical- and occupational therapists, medical doctors, midwifes, porters and radiographs, who responded to both baseline and follow-up questionnaires; yielding a total sample size of 1955 healthcare workers. Furthermore, in subsequent sensitivity-analyses, this study also includes associations specific to participants without LBP at baseline (*n* = 715), as well as to the subpopulation of nurses (*n* = 1180) and nurses without LBP at baseline (*n* = 413). Table 1 shows baseline characteristics of the participants.

### 2.2. Outcome- and Predictor Variables

Intensity of LBP was quantified by the following question:

*“Rate your pain for the low-back within the previous 4 weeks (0–10, where 0 is no pain and 10 is worst imaginable pain).”*

Likewise, with the primary aim of achieving a comprehensive score of overall sleep quality, the following 3 questions adapted from the Bergen Insomnia Scale, were asked:

*“How often during the previous 4 weeks have you…”:*

*(a)*  “awoken during the night and having trouble going back to sleep?”
*(b)*  “been feeling exhausted when waking up?”
*(c)*  “been feeling tired during the day?”

The questions were rated on a 5-point Likert scale with possible answers consisting of “never”, “rarely”, “sometimes”, “often” and “always”. The combined scores were then converted to a scale ranging from 0–100 and categorized in tertiles; hereby representing poor (0–34), moderate (34–65) and good sleep (65–100), respectively. We have previously reported excellent correlations between the 3 questions used in this study and all 6 questions from the Bergen Insomnia Scale (Pearson’s r; 0.94) [24].

### 2.3. Covariates

In the result section, we report fully adjusted associations between subjective ratings of sleep quality and the prospective risk of a 1-point increase in LBP intensity. The present analysis accounts for the following possible confounders: Age, sex, education, LBP at baseline, body mass index, seniority, smoking, physical activity during leisure time, frequency of patient transfer as well as for factors related to the psychosocial work environment; i.e., influence at work and recognition.

### 2.4. Ethics

By agreement with the Danish Data Protection Agency, the National Research Centre for the Working Environment is allowed to register all questionnaire studies in-house. According to Danish law, questionnaire- and register-based studies need neither informed consent nor approval from ethical and scientific committees (Scientific Ethical Committees Act, §14.2). All data was de-identified and analyzed anonymously.

### 2.5. Statistics

Associations between subjective sleep scores at baseline and LBP at follow-up were modelled using cumulative logistic regression (Proc Genmod, SAS, SAS Institute, Cary, North Carolina), i.e. odds ratios (OR’s) express the odds of one point (scale 0–10) difference between the respective groups (moderate vs good sleep and poor vs good sleep, respectively). Analyses were adjusted for the before-mentioned covariates. To account for clustering, hospital department was entered in the ‘repeated subject’ statement. Estimates are provided as OR’s and 95% confidence intervals (CI).

## 3. Results

Table 1 shows baseline characteristics of all healthcare workers as well as the three subgroups used in the sensitivity analyses. In relation to age and seniority, the groups were quite comparable and a general finding was that those free from LBP at baseline reported better sleep scores.

With “good sleep” as reference, this prospective study presents fully-adjusted associations between baseline sleep scores and risk of LBP at follow-up in the full population of healthcare workers (moderate sleep; OR 1.66, 95% CI 1.35–2.04, poor sleep; OR 2.05, 95% CI 1.57–2.69). In the sensitivity analysis including healthcare workers without LBP at baseline, similar associations were observed (moderate sleep; OR 1.88, 95% CI 1.33–2.67, poor sleep; OR 3.16, 95% CI 1.93–5.17). Likewise, in the sub-groups of nurses (moderate sleep; OR 1.85, 95% CI 1.41–2.43, poor sleep; OR 2.40, 95% CI 1.70–3.41) and nurses without LBP at baseline (moderate sleep; OR 1.77, 95% CI 1.12–2.82, poor sleep; OR 2.85, 95% CI 1.48–5.49), respectively, similar dose-response relationships were observed (Table 2).

## 4. Discussion

The present study reports clear dose-response relationships between subjective sleep scores at baseline and risk of LBP at follow-up among healthcare workers, with strong associations found in all sensitivity analyses. Considering the high prevalence of musculoskeletal disorders in this occupational group, the results presented herein constitute a principal present-day perspective towards improving current preventative strategies at the workplace.

In line with recent literature [8,13], the presented findings confirm and extend the potent association between sleep and pain; demonstrating that it unreservedly also holds true for the subgroup of healthcare workers when controlling for various confounders. This notion exemplifies the need for an increased focus on multimodal interventions aiming to decrease the high prevalence of musculoskeletal disorders (MSDs) and would, in the fullness of time, rightfully complement a field of research strongly influenced by a biomechanical perspective [26,27]. Indeed, multimodal implementation strategies—including administrative, environmental and behavioral aspects—have been shown to decrease the incidence of MSDs among healthcare workers where single-mode interventions historically have failed [28,29,30]. Heeding this knowledge in light of the present results, future projects will likely benefit from embracing behavioral approaches targeting not only conceptions and beliefs about pain, but also strategies aiming to elucidate the importance of properly managing relevant lifestyle factors such as stress and sleep.

For reasons largely unknown, the unwavering complexity of both human behavior and pain often seems lost, or at least partially forgotten, within research focusing on improving aspects of the local working environment. This is in stark contrast to the recent (i.e., 15 years) developments within pain research, often referred to as “modern pain science”, where the experience of pain is viewed through a biopsychosocial lens [31,32,33,34]. Following this, the inherent intricacies of pain, especially persistent pain, should be acknowledged as part of an overarching protection system that serves to protect the organism from harm. Therefore, the unconventionally high prevalence of pain within the realm of healthcare likely reflects a scenario in which its habitants are subjected to a wide array of potentially threatening inputs (e.g., mechanical overload, under-staffing, odd working hours, psychosocial stressors and sleep deprivation). However, whereas the majority of these external stressors only become potent when consistent in time, the consequences of even minor sleep deprivation are palpable already the next day [35,36].

Therefore, alongside proper stress management and pain education, it seems that increasing overall sleep quality constitutes a woefully over-looked priority in the current skirmish against pain among healthcare workers. Following this, the present study serves not only to elucidate the aforementioned strong associations, but also to highlight the extent of the issue in this population and hereby shed light upon the largely untapped potential of implementing a biopsychosocial approach towards improving the local working environment in hospitals.

### Strengths and Limitations

Underreporting, non-response and recall bias arguably constitute the primary limitations of utilizing questionnaire surveys [37,38,39]. Combined with the specificity of the population (i.e., healthcare workers and subpopulations sufficiently high in numbers), any endeavors to generalize the results should be approached with caution. Strengths include controlling for a large number of potential confounders, which largely excludes the possibility of the observed associations arising due to confounding. Moreover, the relatively large and homogeneous sample size, as well as the prospective design, arguably give rise to the robustness of the results presented herein. Hospitals with the aim of better managing musculoskeletal health challenges at the workplace are recommended to consider the implementation of organizational initiatives to improve critical lifestyle factors, including sleep hygiene, of the workers.

## 5. Conclusions

Poor sleep constitutes a potent risk factor for LBP among healthcare workers, with strong associations present in all subgroup analyses. The presented results provide strong incentive to evaluate and weigh current prevention policies against an updated biopsychosocial framework, towards creating a healthy and sustainable working environment at hospitals.

## Figures and Tables

**Table 1:** Demographics, work, health and lifestyle variables at baseline.

Variable	All			All, without LBP			Nurses			Nurses, without LBP		
	Mean	SD	%	Mean	SD	%	Mean	SD	%	Mean	SD	%
*N*	1955			715			1180			413		
Female			86.8			85.3			100			100
Age (y)	48.8	10.7		48.8	11.0		48.4	10.4		48.7	10.4	
Years in profession	18.3	11.6		18.3	11.6		18.6	11.4		18.5	11.0	
												
**Sleep quality (0–100)**												
												
Poor sleep (0–34)			24.4			14.8			25.3			14.8
Moderate sleep (34–65)			43.9			43.6			45.0			45.5
Good sleep (65–100)			31.7			41.5			29.7			39.7
												
**LBP intensity at baseline (0–10)**												
												
Poor sleep (*n* = 477)	3.50	2.81					3.43	2.73				
Moderate sleep (*n* = 858)	2.25	2.44					2.25	2.39				
Good sleep (*n* = 620)	1.48	2.02					1.53	2.06				
												
**Education:**												
												
Nurse			61.7			59.2			100			100
Doctor			11.7			15.2						
Healthcare assistant			7.0			6.6						
Physiotherapist			5.1			6.4						
Other			14.5			12.6						

SD—standard deviation; LBP—low-back pain.

**Table 2:** Sleep score and risk of 1-point increase in low-back pain intensity at 1-year follow-up. Values are presented as fully adjusted odds ratios (OR) and 95% confidence intervals (CI) *.

**Sleep Score**	**All**			**All, without LBP at Baseline**		
	**OR**	**95% CI**	***p*-Value**	**OR**	**95% CI**	***p*-Value**
Good (65–100)	1	-	-	1	-	-
Moderate (34–65)	1.66	1.35–2.04	<0.0001	1.88	1.33–2.67	0.0004
Poor (0–34)	2.05	1.57–2.69	<0.0001	3.16	1.93–5.17	<0.0001
**Sleep Score**	**Nurses**			**Nurses, without LBP at Baseline**		
	**OR**	**95% CI**	***p*-Value**	**OR**	**95% CI**	***p*-Value**
Good (65–100)	1	-	-	1	-	-
Moderate (34–65)	1.85	1.41–2.43	<0.0001	1.77	1.12–2.82	0.0153
Poor (0–34)	2.40	1.70–3.41	<0.0001	2.85	1.48–5.49	0.0017

Note: * Adjusted for age, sex, education, LBP at baseline, body mass index, seniority, smoking, physical activity, frequency of patient transfer, influence and recognition at work.

## Data Sharing Statement

Researchers interested in the data should contact the project leader Lars L. Andersen.
